# Study on Surface Quality Analysis of an Uncoated Boron Steel and Its Oxide Layer Suppression Method for Hot Stamping

**DOI:** 10.3390/ma17225563

**Published:** 2024-11-14

**Authors:** Jiho Lee, Junghan Song, Gihyun Bae

**Affiliations:** Flexible Manufacturing R&D Department, Korea Institute of Industrial Technology, Incheon 21999, Republic of Korea; jksg0319@kitech.re.kr (J.L.); jhsong@kitech.re.kr (J.S.)

**Keywords:** hot stamping, boron steel sheet, oxide layer, atmosphere control, induction heating module

## Abstract

This study investigates the effects of hot stamping on boron steel surface properties, comparing uncoated steel to Al–Si-coated steel, with a focus on developing atmosphere-controlled hot stamping technology. Experiments using a hat-shaped specimen revealed that uncoated steel formed a thick oxide layer due to exposure to atmospheric oxygen at high temperatures, negatively impacting surface quality and weldability. In contrast, the Al–Si-coated steel showed no oxide formation. Although uncoated steel exhibited higher average Vickers hardness, the detrimental effects of the oxide layer on weld quality necessitate advancements in process technology. A lab-scale hot stamping simulator was developed to control atmospheric oxygen levels, utilizing a donut-shaped induction heating coil to heat the material above 1000 °C, followed by rapid cooling in a forming die. Results demonstrated that maintaining oxygen concentrations below 6% significantly reduced oxide layer thickness, with near-vacuum conditions eliminating oxide formation altogether. These findings emphasize the critical role of oxygen control in enhancing the surface quality and weldability of uncoated boron steel for ultra-high-strength automotive applications, potentially reducing manufacturing costs while ensuring part performance.

## 1. Introduction

Due to recent environmental concerns and strengthened fuel efficiency regulations, the demand for eco-friendly vehicles is increasing. Many global automotive companies are producing electric vehicles as a means to satisfy various environmental regulations by replacing traditional internal combustion engines. In order to achieve maximum driving range with a single battery charge, the weight of the vehicle has become crucial for environmentally friendly vehicles. To reduce vehicle weight, many companies are increasing the application of hot stamping components to ensure collision performance and passenger safety. As evidence of this, after the initial application of hot stamping components made of boron steel in a Saab vehicle in 1984 [1], the production ratio of hot stamping increased from 3 million to 8 million in 1987 and further surged to 170 million in 2007 [2]. In 2023, the hot stamping market is expected to expand to approximately 1.28 billion components. Accordingly, research related to hot stamping production technology is actively being conducted.

The hot stamping method is suitable for producing high-strength products required for passenger safety by forming the material with high elongation and low strength under a high temperature of over 900 °C and then quenching it to the martensitic steel in order to achieve very high strength over 1.5 GPa. The quenching process involves rapid cooling at speeds exceeding approximately 27 K/s from the martensite transformation temperature, ranging from 425 °C (martensite start point) to 280 °C (martensite finish point) [3]. Materials commonly used for hot stamping include alloys with boron additions such as 22MnB5, 27MnB5, and 37MnB5. Boron-added alloys are preferred in hot stamping, as boron steel is known to be the only type of steel capable of generating a martensitic microstructure after hot stamping [4]. Boron steel sheets typically have tensile strength levels of around 600 MPa before heat treatment. Through the martensitic transformation of the material after hot stamping, it is known to achieve tensile strengths of approximately 1.5 GPa [4].

Numerous researchers have investigated component manufacturing technologies utilizing uncoated hot stamping steel sheets. From a materials technology perspective, various experimental analyses have been conducted to ensure and enhance the mechanical properties of materials, with the cooling rate selected as a key variable [5,6,7]. Concurrently, from a process technology perspective, researchers have focused on optimizing the hot stamping process to improve forming quality and productivity [8,9,10,11,12]. Furthermore, a variety of studies have been conducted to analyze heat transfer and material deformation mechanisms in the hot stamping process, aiming to enhance process efficiency [13,14,15,16]. These collective studies have continuously advanced component manufacturing technologies that utilize uncoated hot stamping steel sheets.

Hot-stamped boron steels without Al–Si coating layer generate an oxide scale on the surface when exposed to air under austenitizing conditions. To control this oxide layer, methods such as using boron steel sheets coated with Al–Si for oxidation prevention [17] or applying oil for anti-oxidation on the surface [18] can be employed. Physical methods involving shot blasting can also remove the oxide layer [18,19]. While these methods offer the advantage of controlling the oxide layer during hot stamping, the presence of a coating layer on the surface for oxidation control can lead to the melting of the coating layer during welding. Additionally, when using oil for oxidation prevention, research data indicates a decrease in interfacial heat transfer coefficients (IHTC), affecting the microstructure and mechanical properties [20]. The Al–Si coating layer, when subjected to rapid heating rates, can experience premature heating, causing the coating layer to slide and leading to issues such as coating layer displacement. Moreover, the production cost of hot-stamped parts should be increased due to material and process patents related to Al–Si-coated boron steel sheets.

To overcome these drawbacks, it is important to suggest an alternative hot stamping method by using an uncoated boron steel sheet. Taylor et al. [21] illustrated a schematic of the hot stamping process for uncoated steel sheets, demonstrating that this process generally includes an additional shot blasting step to remove the oxidation scale. This additional step requires extra production equipment and extends the processing time, resulting in increased manufacturing costs for hot-stamped parts using uncoated steel sheets. The comparatively higher manufacturing cost, as opposed to coated steel sheets, presents a significant obstacle to the use of non-coated steel sheets. Consequently, even with the additional costs associated with paying for material and process patents held by Arcelor Mittal, most automotive body component manufacturers opt to use Al–Si-coated steel sheets. Chen et al. [22] emphasized the cost-saving advantages of uncoated hot stamping processes in automotive part manufacturing. Additionally, Kim et al. [23] demonstrated through an economic efficiency analysis that using uncoated steel sheets in hot stamping processes can reduce production costs and improve efficiency. Therefore, to reduce the manufacturing cost of hot-stamped components, automotive companies and body component manufacturers are seeking the development of press-forming technology that can suppress the formation of oxide layers on non-coated steel sheets. It means that preventing or controlling oxide layer growth should be a core technology for using uncoated boron steel sheets.

This paper deals with the quantitative investigation of the surface quality of a hot-stamped part and its improvement method of the oxide layer for uncoated boron steel sheet. Initially, the heat treatment results with hat-type specimen were compared according to the presence of the Al–Si coating layer in order to observe the effect of the coating layer on the surface quality and mechanical properties. To investigate methods for suppressing the oxide layer on uncoated steel sheets, a lab-scale hot stamping simulator was developed to replicate the heating and forming processes under controlled atmospheric conditions. Subsequently, the oxide layers were quantitatively analyzed according to the oxygen ratio. The research results can be utilized as hot stamping process control technology to ensure surface quality and weldability in the manufacturing process of ultra-high-strength body components using uncoated steel sheets.

## 2. Hot Stamping Test Method

### 2.1. Equipment Setup

Al–Si-coated boron steel is known to prevent the formation of an oxide layer by blocking the oxygen contact during the hot stamping process [17]. For using uncoated boron steel sheets, the surface quality and property should be investigated to observe the oxide layer formation and to ensure the mechanical properties of hot-stamped parts.

**Figure 1:**
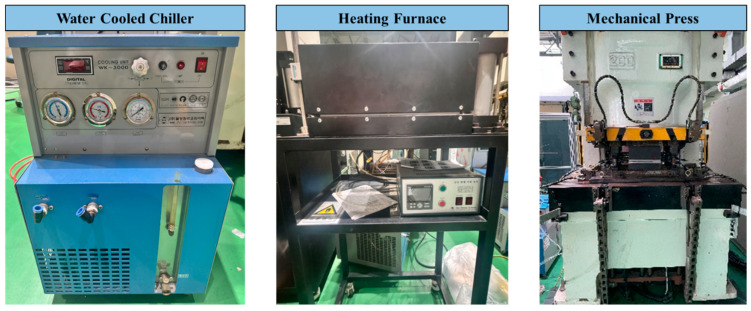
Main test devices for the hot stamping test.

An experimental setup, as shown in Figure 1, is constructed in order to reproduce the hot stamping process. An atmosphere furnace has the capability of heating up to approximately 1000 °C, and a mechanical press can make hat-type specimens by using a hot stamping die. A water-cooling chiller is connected to the stamping die for rapid cooling, and a thermometer is used to monitor the temperature at the stamping die. The 300 mm × 300 mm sample was heated up to 950 °C and stabilized for 10 min. After that, they rapidly cooled down to 10 °C or below within 5 s for the martensite phase transformation in the die. 22MnB5 1.0 t steel sheets, produced by Arcelor Mittal, with and without Al–Si coating layer, were used for experiments. The chemical composition and the mechanical strength of 22MnB5 base metal are presented in Table 1.

### 2.2. Test Procedure

**Figure 2:**
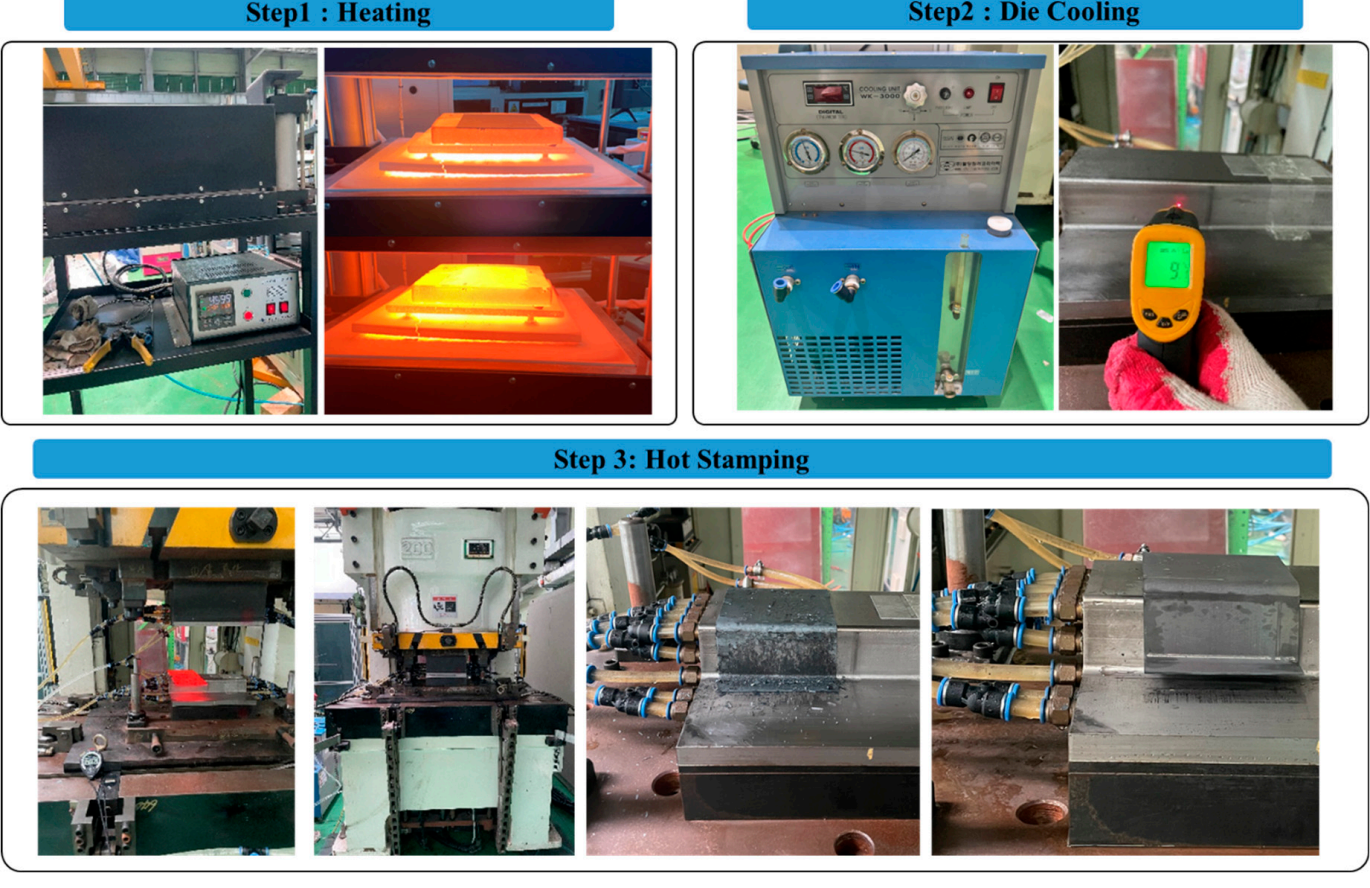
Hot stamping test process of a hat-type specimen.

To observe the surface property changes after hot stamping for both uncoated and Al–Si-coated materials, specimens were subjected to the same heat treatment conditions. The specimens were heated by the controllable atmosphere furnace up to 950 °C and stabilized for 10 min. Subsequently, they were quenched and formed simultaneously using a 200-ton servo press with a water-cooled die that was pre-cooled to below 10 °C. The time taken for the material to reach the press was approximately 5 s. The detailed test procedure is presented in Figure 2.

## 3. Hot Stamping Test Results

### 3.1. Surface Investigation

**Figure 3:**
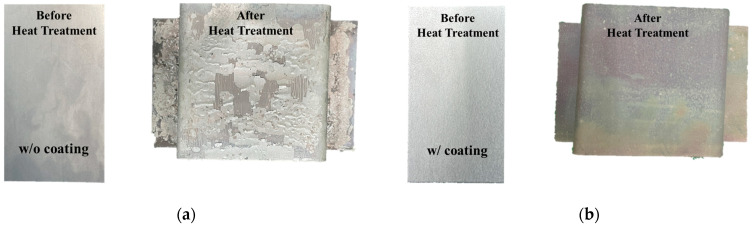
Hat-type specimens before and after the hot stamping test: (**a**) uncoated material; (**b**) coated material.

**Figure 4:**
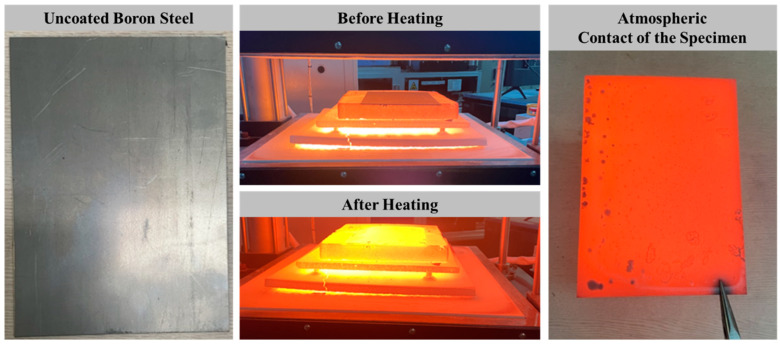
Oxide layer formation of the uncoated boron steel due to oxygen contact.

Hot stamping experiments were performed with hat-type specimens in order to observe the growth of the oxide layer and to investigate the surface property of the coated and uncoated boron steel sheet. Figure 3 shows test specimens according to the coating layer. The specimen with Al–Si coating layer shows only the heat-affected color change due to the rapid temperature change during the heating and quenching process, and no oxide layer was observed as shown in Figure 3a. This aligns with the known characteristic of Al–Si coating preventing the formation of oxide layers by blocking contact with the atmosphere after heating [17]. As shown in Figure 4, the heated material generates an oxide layer due to contact with oxygen in the air during the transport process, and after rapid cooling during the component forming process, a thick oxide layer exists as shown in Figure 3b.

### 3.2. Oxide Layer

**Figure 5:**
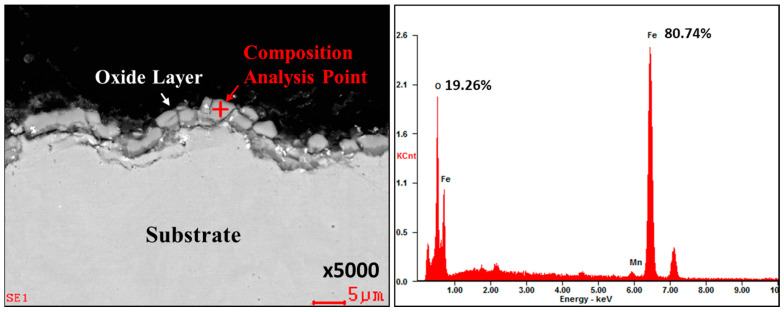
Point EDS composition analysis of uncoated boron steel sheets after heat treatment.

For a more precise analysis of surface layer changes, the SEM (scanning electron microscope) equipment was employed to observe coated and uncoated boron steel sheets before and after heating. The equipment used for observations is the QUANTA 200F (FEI Company, Hillsboro, OR, USA). First of all, point EDS measurements were carried out to investigate the composition of the surface scale of the uncoated boron steel sheet. Figure 5 shows the result of point EDS measurements of the surface scale. The composition of the surface scale was Fe 80.74 wt% and O 19.26 wt% components, and it reveals that the surface scale is the oxide layer of Fe and O.

**Figure 6:**
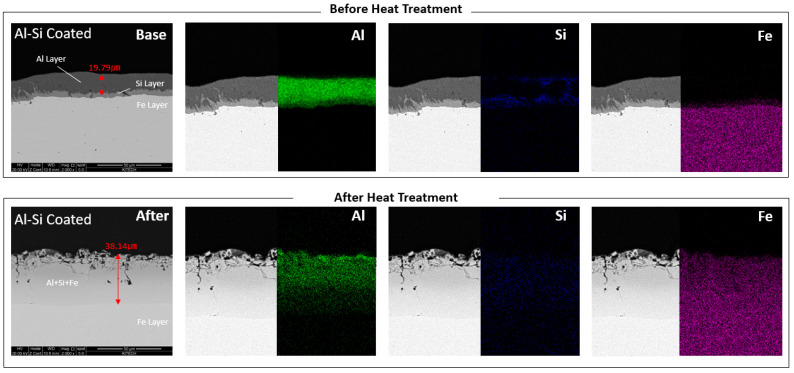
EDS mapping analysis of Al–Si-coated boron steel sheets before and after heat treatment.

The Al–Si-coated specimen was analyzed using EDS mapping in order to investigate the change in the composition at the coating layer before and after heat treatment. As shown in Figure 6, Al–Si coating layer is transformed into the mixed intermetallic layer of Al–Si and Fe after the heat treatment. As shown in Figure 6, fine cracks and internal voids are observed in the rough intermetallic layer, which are generated during the rapid quenching process. Such compounds are known to have low fracture toughness, promoting the formation and growth of fine cracks [24,25]. As a result of the diffusion and alloying of components, the coating thickness is increased from 19.79 μm to 38.14 μm after heat treatment.

**Figure 7:**
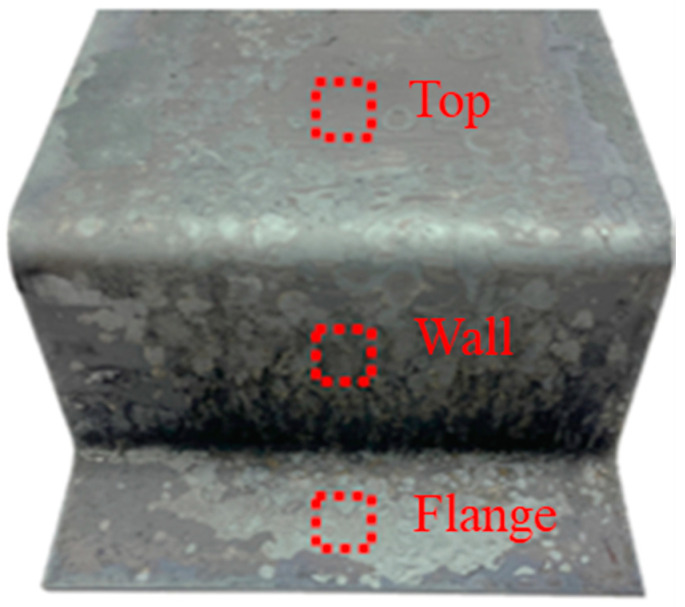
Sample-capturing locations of the hat-type specimen.

**Figure 8:**
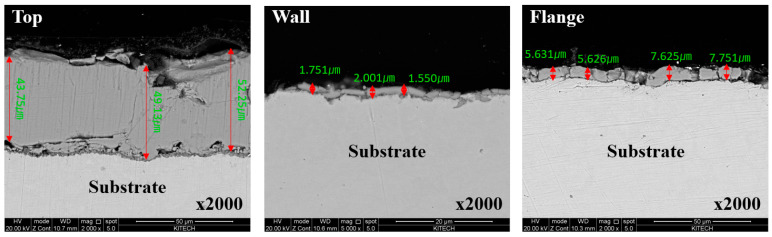
SEM images at the top, wall, and flange locations to measure the oxide layer thickness of uncoated boron steel.

The oxide layer thickness was quantitatively measured by optical microscope with a hat-type specimen of uncoated boron steel sheet in order to investigate the oxide layer residual due to the different contact mechanisms between die and sheet during the hot stamping. Figure 7 shows the captured location of samples from the hat-type uncoated specimen. As shown in Figure 8, the oxide layer thickness was 23.46 μm, 1.81 μm, and 7.55 μm at the top, wall, and flange locations, respectively. At the top location, the thickest oxide layer was observed due to no contact between the die and the sheet. On the contrary, most of the oxide layer was removed in the wall location because severe contact occurs when the sheet slides against the die during the forming. At the flange location, the thickness is decreased due to the high compression of the die at the bottom dead center of the press slide. Most spot welds are made at the top and flange areas in the auto-body assembly process. It means that the thick oxide layer can deteriorate the welding quality by using an uncoated boron steel sheet in the hot stamping. Therefore, a novel stamping method is required to reduce or control the oxide layer formation in order to utilize the uncoated boron steel sheet for ultra-high-strength automotive parts.

**Figure 9:**
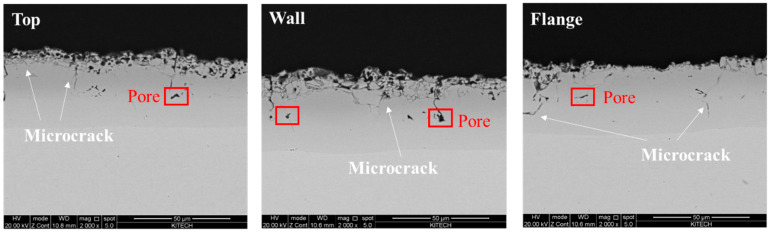
SEM images of Al–Si-coated hat-type specimen at the top, wall, and flange locations.

The optical microscope images of Al–Si-coated specimen are presented in Figure 9. Because of the Al–Si coating layer, increased coating thickness according to the diffused alloying components is observed instead of the oxide layer. Additionally, microcracks and pores are present within the coating due to the different coefficients of thermal expansion [25,26,27]. No significant difference was observed at the top, wall, and flange locations due to the contact pressure during the forming process.

### 3.3. Hardness

**Figure 10:**
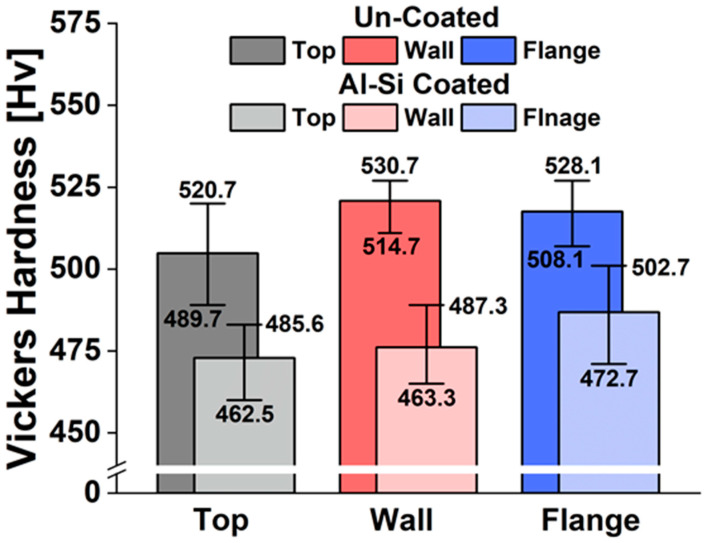
Vickers hardness of uncoated and coated boron steel sheets.

A Vickers hardness test was conducted to examine the effect of the absence of a coating layer on material strength during the forming process of uncoated steel sheets. Hardness is generally known to correlate with material strength. Kim et al. [28] reported that the Vickers hardness of a boron steel sheet is approximately 450 Hv after the hot stamping thermal cycle. Based on phase transformation tests, Li et al. [29] demonstrated that the Vickers hardness of boron steel is 459 Hv at a cooling rate of 30 °C/s and that the hardness increases proportionally with the rise in cooling rate. In this paper, Vickers hardness was measured using a SHIMAZU HMV-2R (Shimadzu Corporation, Kyoto, Japan) device under the condition of applying a load of 980.7 mN for 10 s. Figure 10 shows the Vickers hardness measurement results for both coated and uncoated steel sheets. To examine the effect of cooling pattern changes due to differences in contact conditions between the material and the die, hardness was measured in the top, wall, and flange areas. The hardness was measured seven times at 1 mm intervals. The overall average hardness of the coated and uncoated steel sheets was measured at 481 Hv and 513 Hv, respectively, with higher values observed for the uncoated steel sheets. Therefore, it was confirmed that uncoated materials are more advantageous for strength securement in hot stamping, which requires rapid cooling. The hardness differences at various measurement locations were not considered significant when taking into account the scatter in the data, suggesting that the impact of the contact conditions between the die and the material on hardness is minimal. Despite the differences due to the presence or absence of a coating layer and contact conditions, the hat-type specimen can achieve the desired tensile strength in all areas.

### 3.4. Summary

For the boron steel with Al–Si coating, as expected, no oxide layer was observed on the surface during the heat treatment and hot stamping processes. However, microcracks and pores were identified within the coating layer due to the different thermal expansion coefficients. Additionally, the elements clearly distinguished in the coating layer before heat treatment diffused during the heat treatment, forming a Fe_3_(AlSiFe) alloy layer inside the coating layer, which could potentially compromise the weldability. The coating layer thickness also increased approximately two-fold. On the other hand, the uncoated boron steel exhibited irregular and uneven oxide layers on the surface when exposed to the atmosphere during the heat treatment process. Hot stamping simulation tests revealed that the oxide layer thickness could be preserved, peeled off, or compressed according to the die shape. The oxide layer thickness was observed to be over 20 μm, which could cause weldability issues in the assembly process of automotive parts. The use of uncoated steel sheets is expected to be advantageous for securing material strength.

During the hot stamping process using uncoated 22MnB5 material, the oxidation reaction was most pronounced at the point of material transfer. This observation indicated that the material, upon being heated and transferred, came into contact with atmospheric oxygen, leading to the initiation and rapid growth of the oxide layer. Therefore, to ensure the surface quality of uncoated 22MnB5 material during hot stamping, controlling the contact between the heated material and oxygen during transfer is crucial for maintaining surface quality. Therefore, further research on new forming technologies is needed to suppress the formation of oxide layers in order to ensure the surface quality and weldability of uncoated steel sheets.

## 4. Oxide Layer Improvement

### 4.1. Development of a New Hot Stamping Simulator

The issue of oxide layer formation during the hot stamping process of uncoated steel sheets occurs because the material rapidly reacts with oxygen in the atmosphere at high temperatures, leading to the formation of an oxide layer. In particular, controlling the oxygen concentration in the atmosphere is crucial to suppressing the formation of the oxide layer. Developing a new lab-scale simulator that can replicate the hot stamping process is a very important approach to achieve this.

**Figure 11:**
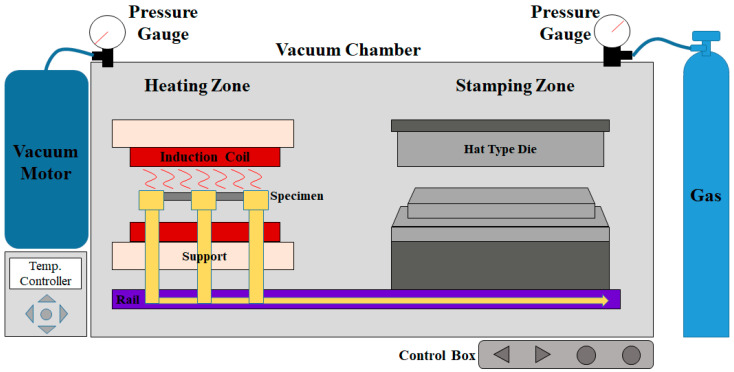
Schematic diagram of an atmosphere-controllable hot stamping simulator.

A lab-scale simulator allows for a quantitative analysis of the oxide layer thickness under various conditions, such as oxygen concentration and temperature, providing critical data for process optimization. Figure 11 shows the schematic diagram of the hot stamping simulator. The experimental equipment consists of a heating zone and a forming zone, including a transfer system for continuous forming after heating. To control the oxygen content in the atmosphere during the experiment, the entire equipment is sealed within a vacuum chamber. A nitrogen gas injection system was introduced to enhance the efficiency of atmospheric control. With this configuration, the vacuum chamber and nitrogen gas injection system allow the setting of controlled atmospheric conditions, making it possible to simulate the hot stamping process of uncoated steel sheets through the heating-transfer-forming sequence.

**Figure 12:**
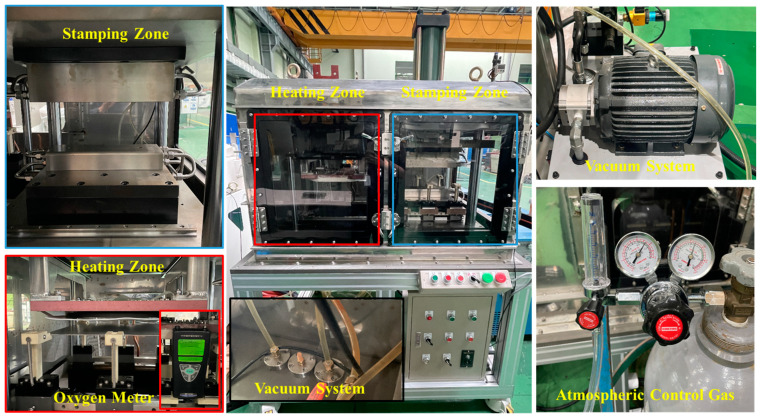
Lab-scale hot stamping simulator.

Figure 12 shows the manufactured experimental equipment. In the heating zone, a 300 mm × 300 mm specimen is placed on a transfer jig and heated using a pancake-shaped induction coil installed above it. Once the specimen reaches the target temperature, it is transferred to the stamping zone via a transfer device operated by a mechanical motor, where it is then formed by an upper die driven by a hydraulic cylinder. During this process, both the upper and lower dies are cooled with water, creating rapid cooling conditions that promote martensitic structure formation. For oxidation layer control experiments, an atmosphere control system was implemented as previously described, using a vacuum system to evacuate air from the chamber while injecting nitrogen gas to quickly reach the desired oxygen level. Using this newly developed hot stamping simulator, forming tests on non-coated hot stamping steel sheets were conducted at different oxygen levels to study the potential for oxidation layer suppression.

### 4.2. Establishment of the Experimental Condition

**Figure 13:**
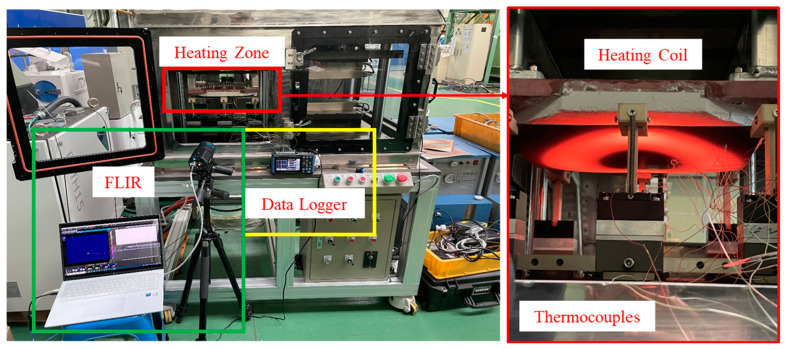
Equipment configuration for the performance evaluation of the heating module.

**Figure 14:**
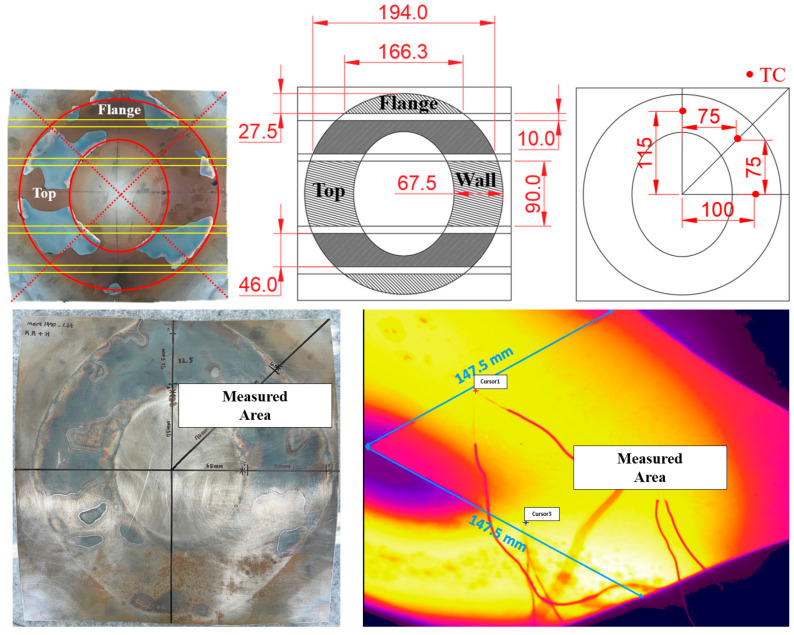
Locations of thermocouple attachment considering the heated area.

The simulator equipment uses a pancake-shaped induction coil to heat material of 300 mm × 300 mm size to temperatures above 950 °C. Due to the shape of the coil and the thermal deformation of the specimen, temperature variations occur at different positions. Therefore, a heating test was conducted to select the optimal output of the heating module for achieving the target temperature and to determine the analysis area for surface layer examination based on temperature variations. To measure the temperature distribution of the specimen, a FLIR A700 (FLIR Systems, Wilsonville, OR, USA) infrared camera capable of measuring temperatures up to 2000 °C was used. Figure 13 shows the heating performance test of the material using the FLIR camera. First, the material was heated under a 12 kW condition, and Figure 14 illustrates the distribution of the oxide layer formed after the heating test. Through this preliminary heating test, it was confirmed that the material was heated in line with the shape of the coil and that the oxide layer formed accordingly. The FLIR camera was used to select 1/4 of the dome-shaped specimen for measurement, considering the measurement range. Three thermocouples were attached to the wall section and flange section to measure the reference temperature for calibration.

**Figure 15:**
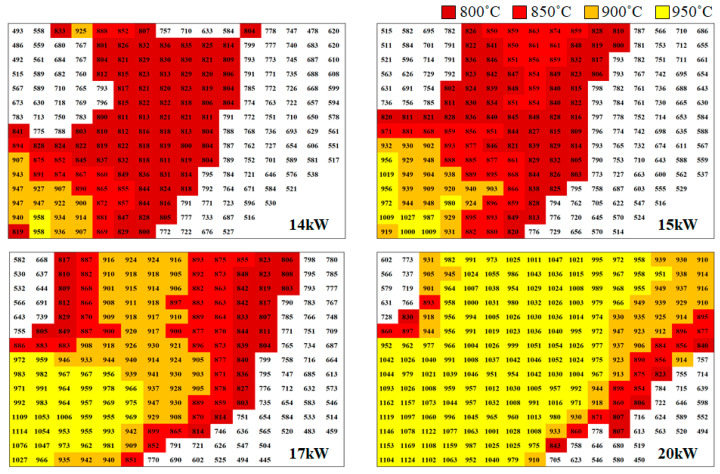
Temperature distribution according to the heating module output.

Afterward, heating tests were conducted according to the output of the heating module, and the temperature distribution was observed using the FLIR camera. Figure 15 shows the temperature distribution map corresponding to the heating module output. Since the target temperature for the hot stamping process is above 950 °C, this area was marked in yellow on the temperature distribution map. Through the heating test, it was confirmed that an output of 20 kW is required to maintain the target temperature across a wide area of the hat-type specimen. Therefore, the optimal output for forming tests under controlled oxygen conditions was set to 20 kW.

**Figure 16:**
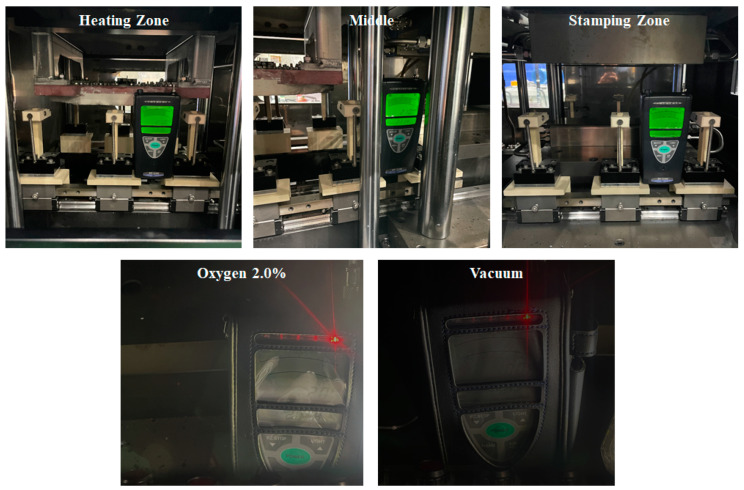
Installation of the oxygen analyzer inside the vacuum chamber.

**Figure 17:**
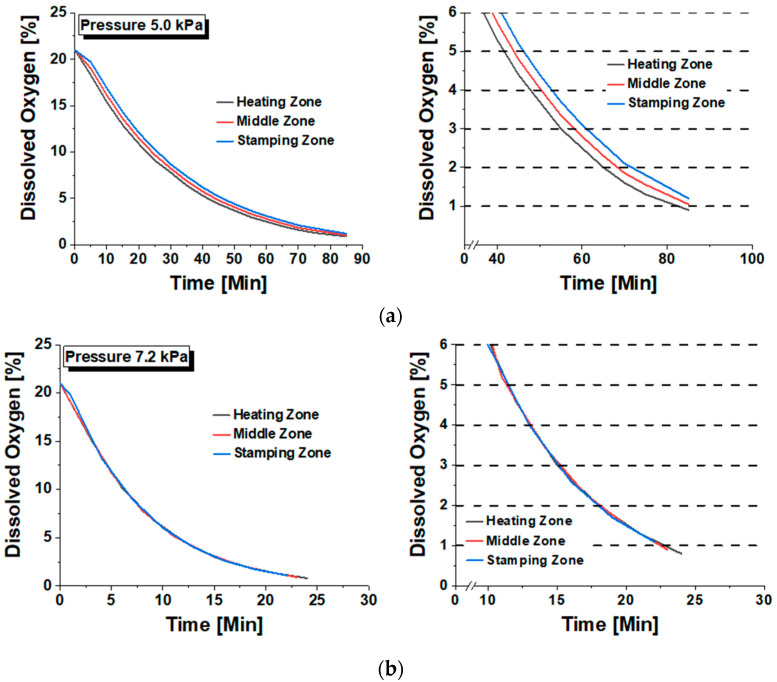
Atmosphere control test result according to the nitrogen gas pressure: (**a**) 5.0 kPa; (**b**) 7.2 kPa.

To verify the atmosphere control performance of the vacuum chamber, the changes in oxygen content according to nitrogen gas pressure were observed. The oxygen content was controlled by simultaneously removing air from the chamber using a vacuum pump and injecting nitrogen gas. To check the oxygen level inside the chamber, an oxygen analyzer was installed as shown in Figure 16. The oxygen analyzer was moved between the heating section, transfer section, and forming section to measure the changes in oxygen content over time, ensuring uniformity in the oxygen distribution. Figure 17 shows the history of oxygen content changes according to nitrogen gas pressure and time. It was confirmed that the oxygen content could be controlled to below 1% over time. However, when the nitrogen pressure was low at 5 kPa, it took over 80 min to control the oxygen content to below 1%, and non-uniformity in oxygen distribution across different positions was observed. To improve this, when the nitrogen gas pressure was increased to 7.2 kPa, the time required to achieve the same level of oxygen control was reduced to under 25 min, and the oxygen distribution became uniform across the chamber. Therefore, it is considered that a nitrogen gas pressure of 7.2 kPa is appropriate for atmosphere control.

### 4.3. Evaluation of Oxide Layer

**Figure 18:**
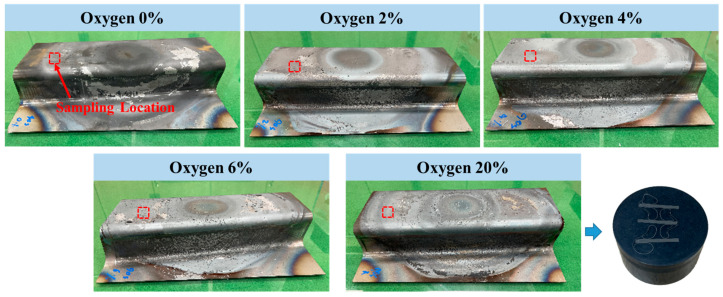
Experimental results of simulated specimens through atmosphere control.

Forming tests on hat-type specimens made of uncoated steel sheets were conducted using a lab-scale hot stamping simulator, with heating and pressure control, to examine the effects of oxygen levels. After stabilizing the atmosphere according to the oxygen level, the material was heated by an induction coil, transferred to the hot stamping die by an automatic transfer device, and then formed into a hat-type specimen inside the die using water cooling. To observe the amount of oxide layer formation based on oxygen levels, forming tests were conducted under oxygen conditions of 0% (vacuum-like), 2%, 4%, 6%, and 21% (atmosphere), as shown in Figure 18. Under the vacuum-like condition, almost no oxide layer was observed. However, as the oxygen level increased, the amount of oxide layer also gradually increased, and at 6% oxygen, the oxide formation was almost similar to that observed under atmospheric conditions.

**Figure 19:**
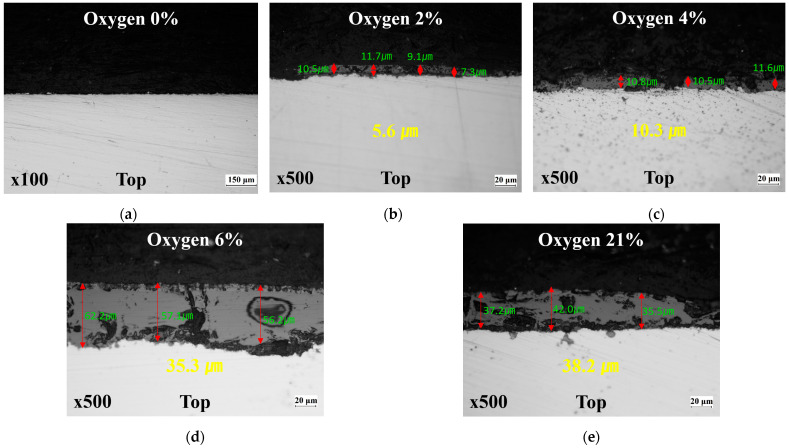
Optical microscope measurements according to atmosphere conditions at the top area: (**a**) oxygen 0%; (**b**) oxygen 2%; (**c**) oxygen 4%; (**d**) oxygen 6%; (**e**) oxygen 8%.

For a more quantitative observation, samples were taken from the top area under different oxygen conditions because the top area is the most susceptible to oxide layer formation. The thickness of the oxide layer was measured using an optical microscope. The equipment used for observations is the ECLIPSE MA22 (Nikon, Tokyo, Japan). Figure 19 shows the optical microscope images for each condition. When the amount of oxygen is close to a vacuum condition, almost no oxide layer forms. As the oxygen level gradually increases, the thickness of the oxide layer also increases proportionally, and at an oxygen level of 6%, an oxide layer similar to that formed under atmospheric conditions is observed. This shows that the oxide layer of uncoated boron steel is highly sensitive to the amount of oxygen.

In the case of typical automotive parts, body assembly is carried out through processes such as spot welding after hot stamping. At this time, as the thickness of the residual oxide layer increases, the quality of welding can deteriorate rapidly. Therefore, for the hot stamping of uncoated boron steel, it is necessary to control the atmosphere to minimize the amount of oxygen in the environment during formation. Otherwise, surface oxide layer removal through processes such as shot peening will be required, which increases manufacturing costs and becomes an obstacle to using uncoated boron steel sheets. However, in actual component manufacturing processes, controlling the oxygen level close to a vacuum to suppress the oxide layer is highly challenging. It is more practical to introduce a threshold oxygen level condition in the hot stamping process of uncoated steel that ensures welding quality. Future research should focus on determining the optimal oxygen control conditions by analyzing the correlation between oxide layer formation and welding quality.

## 5. Conclusions

This study aimed to compare and analyze the surface property changes of boron steel during the hot stamping process, depending on the presence or absence of Al–Si coating, and to develop an atmosphere-controlled hot stamping technology for applying uncoated boron steel to ultra-high-strength automotive parts. Hot stamping experiments using a hat-shaped specimen showed that while no oxide layer formed on the Al–Si-coated steel, a thick oxide layer was observed on the uncoated steel due to exposure to atmospheric oxygen at high temperatures. This oxide layer not only deteriorates the surface quality of the uncoated steel but also significantly hinders weldability during the assembly of automotive parts. Surface hardness analysis revealed that the Vickers hardness of uncoated steel was, on average, 32 Hv higher than that of Al–Si-coated steel, suggesting that uncoated steel may be more advantageous for securing part strength. However, the formation of a thick oxide layer poses a significant risk to weld quality, making process technology to suppress oxide formation essential for the practical application of uncoated steel.

To address this issue, a new lab-scale hot stamping simulator was developed. This equipment was designed to perform forming experiments in an environment where the amount of oxygen in the atmosphere could be controlled to suppress oxide layer formation. The system uses a donut-shaped induction heating coil to heat the material to over 1000 °C and quickly transfer it to a forming die for rapid cooling and shaping through an automated transfer system. The oxygen concentration in the simulator was controlled using nitrogen gas and a vacuum pump, allowing the analysis of oxide layer formation under various oxygen levels. The experiment results showed that when the oxygen level in the atmosphere was below 6%, the thickness of the oxide layer decreased significantly, and in near-vacuum conditions, almost no oxide layer formed. These findings indicate that controlling the oxygen concentration can effectively suppress oxide layer formation in uncoated steel, improving both surface quality and weldability.

In conclusion, this study confirms that for uncoated boron steel to be applied in ultra-high-strength automotive parts, effective oxygen control during the hot stamping process is essential. By implementing such technology, it is expected that manufacturing costs can be reduced while simultaneously ensuring the strength and weld quality of uncoated steel parts.

## Figures and Tables

**Table 1:** Chemical compositions and mechanical strength of 22MnB5 base metal.

Chemical Compositions [wt%]							Mechanical Strength [MPa]	
C	Mn	B	Si	P	S	Cr	Yield Stress	Tensile Stress
0.24	1.10	0.003	0.26	0.014	0.001	0.14	1254	1545

## Data Availability

The original contributions presented in the study are included in the article, further inquiries can be directed to the corresponding author.
